# Wound Healing Effect of Essential Oil Extracted from *Eugenia dysenterica* DC (Myrtaceae) Leaves

**DOI:** 10.3390/molecules24010002

**Published:** 2018-12-20

**Authors:** Sandra Márcia Mazutti da Silva, Claudio Rodrigues Rezende Costa, Guilherme Martins Gelfuso, Eliete Neves Silva Guerra, Yanna Karla de Medeiros Nóbrega, Sueli Maria Gomes, Aline Pic-Taylor, Yris Maria Fonseca-Bazzo, Damaris Silveira, Pérola de Oliveira Magalhães

**Affiliations:** 1 Natural Products Laboratory, School of Health Sciences, University of Brasília, 70910-900, Campus Universitário Darcy Ribeiro, 70910-900 Brasília-DF, Brazil; sandra.mazutti@hotmail.com (S.M.M.d.S.); yannanobrega@gmail.com (Y.K.d.M.N.); yrisfonseca@unb.br (Y.M.F.-B.); damaris@unb.br (D.S.); 2 Laboratory of Oral Histopathology, Health Sciences Faculty, University of Brasília, 70910-900 Brasília, Brazil; claudim_odonto@yahoo.com.br (C.R.R.C.); elieteneves.unb@gmail.com (E.N.S.G.); 3 Laboratory of Food, Drugs and Cosmetics (LTMAC), School of Health Sciences, University of Brasília, 70910-900 Campus Universitario Darcy Ribeiro, Brasília-DF 70910-900, Brazil; gmgelfuso@unb.br; 4 Department of Botany, Institute of Biological Sciences, University of Brasília, Brasília, Campus Universitário Darcy Ribeiro, Brasília-DF 70910-900, Brazil; smgomes@unb.br; 5 Laboratory of Embryology and Developmental Biology, Genetics and Morphology Department, Institute of Biological Sciences, University of Brasília, 70910-900, Campus Universitário Darcy Ribeiro, Brasília-DF 70910-900, Brazil; alinepic@unb.br

**Keywords:** essential oil of *Eugenia dysenterica*, wound healing, skin cell migration

## Abstract

The use of natural oils in topical pharmaceutical preparations has usually presented safe agents for the improvement of human health. Based on research into the immense potential of wound management and healing, we aimed to validate the use of topical natural products by studying the ability of the essential oil of *Eugenia dysenterica* DC leaves (oEd) to stimulate in vitro skin cell migration. Skin cytotoxicity was evaluated using a fibroblast cell line (L929) by MTT assay. The oil chemical profile was investigated by GC-MS. Moreover, the inhibition of lipopolysaccharide (LPS) induced nitric oxide (NO) production in the macrophage cell line (RAW 264.7) tested. The Chick Chorioallantoic Membrane (CAM) assay was used to evaluate the angiogenic activity and irritating potential of the oil. The oEd induces skin cell migration in a scratch assay at a concentration of 542.2 µg/mL. α-humulene and β-caryophyllene, the major compounds of this oil, as determined by GC-MS, may partly explain the migration effect. The inhibition of nitric oxide by oEd and α-humulene suggested an anti-inflammatory effect. The CAM assay showed that treatment with oEd ≤ 292 µg/mL did not cause skin injury, and that it can promote angiogenesis in vivo. Hence, these results indicate the feasibility of the essential oil of *Eugenia dysenterica* DC leaves to developed dermatological products capable of helping the body to repair damaged tissue.

## 1. Introduction

Wound healing is a multiphase process involving the interaction of a complex cascade of cellular and biochemical stages, leading to the restoration of structural and functional integrity of damaged tissues. This process involves continuous cell-cell interaction of soluble mediators and extracellular matrix interactions that allow the process to proceed in different overlapping phases. The healing process includes inflammation, wound contraction, re-epithelialization, tissue remodeling, and granulation tissue formation with angiogenesis. Therefore, the primary goals of wound treatments are rapid closure, together with a functional and aesthetically satisfactory scar [1].

Nowadays, wound healing poses a significant problem to health-care systems worldwide, since it is estimated that 1–1.5% of the population will have a serious problem with wounds [2]. Diabetes, for example, is a condition that can significantly affect the healing process by altering cell membrane structure and the inflammatory response due to changes in the chemotactic and phagocytic function of white blood cells. Therefore, the blood supply to the injury is reduced, as is angiogenesis, resulting in an ischemic microenvironment [3]. Due to the increased life expectancy of the population, together with a consequent increase in the prevalence of diseases that accompany aging, such as diabetes, the expenses associated with wound treatments has gained attention. In this scenario, it is important to innovate with effective, safe, and more economical alternative wound treatments. Therefore, natural products can emerge as promising candidates.

Myrtaceae is a family of plants found in the Cerrado (savannah biome), which provides products that are used for both alimentary and medicinal purposes. *Eugenia dysenterica* DC (synon. *Stenocalyx dysentericus* Berg., *Myrtus dysenterica* M.), popularly known as “cagaita” or “cagaiteira”, is one important species of this family. According to traditional knowledge, cagaita leaves have anti-diarrheal properties and are also useful for treating diabetes [4]. Moreover, the cagaita fruit has been used in various preparations such as jams, ice-cream, liqueurs, and juices as it provides a source of bioactive nutritional compounds. Several macro and microelements such as vitamins, folates, carotenoids, and phenolic compounds have been identified in the pulp, providing both nutritional and economic benefits [5].

Sesquiterpenes, saponins, and considerable levels of polyphenolic compounds, such as flavonoids (catechin, quercetin, epicatechin ellagic acid) and tannins [6], have also been identified in *Eugenia dysenterica* leaves, which suggests a potential ability to accelerate wound healing processes [7]. The objective of this study was to evaluate the efficacy of the essential oil from *Eugenia dysenterica* DC leaves (oEd) in wound healing and establish a safety profile for human skin cell exposure, with the aim of using it in novel natural treatments for skin injuries in the future.

## 2. Results and Discussion

### 2.1. In Vitro Studies

#### 2.1.1. Essential Oil Yields and Chemical Composition

**Figure 1:**
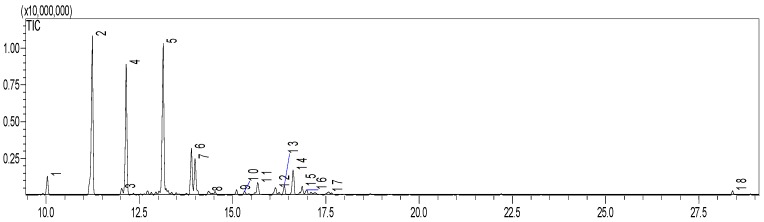
Gas chromatogram of essential oil from *Eugenia dysenterica* leaves. Peak: **1** *(*α-copaene); **2** (E-caryophyllene); **3** (spirolepechinene); **4** (α-humulene); **5** (δ-selinene); **6** (selinene <7-epi-α->); **7** (δ-cadinene); **8**, **9** and **10** (unidentified); **11** (caryophyllene oxide); **12** (humulol); **13** (humulene epoxide II); **14** (7(11)-Selinen-4α-ol); **15** (eudesma-4(15),7-dien-1β-ol); **16** and **17** (unidentified); and **18** (Phytol).

The *Eugenia dysenterica* leaf essential oil was composed mainly of sesquiterpenes (43.39%), in which α-humulene (19.3%) and β-caryophyllene (24.36%) were identified as the major compounds (peaks 2 and 4, respectively) by gas chromatography (Figure 1), thereby corroborating a previously published result [8]. Similarly, the presence of selinene in the oil composition was verified for the first time, which may be related to multifactorial ecological causes [9].

Additionally, a GC-MS analysis was carried out for quantification of α -humulene in the sample. A standard curve of α-humulene was used to determine the concentration of this compound. α-humulene was found at a concentration of 0.01 mg/mL in *Eugenia dysenterica* essential oil.

#### 2.1.2. Cytotoxicity-Tetrazolium-Based Colorimetric Assay (MTT)

Cell viability was evaluated to determine the cytotoxic potential of essential oil and α-humulene in epithelial lineages, which in the latter case could provide valuable information about the safety of these active compounds when topically applied.

**Figure 2:**
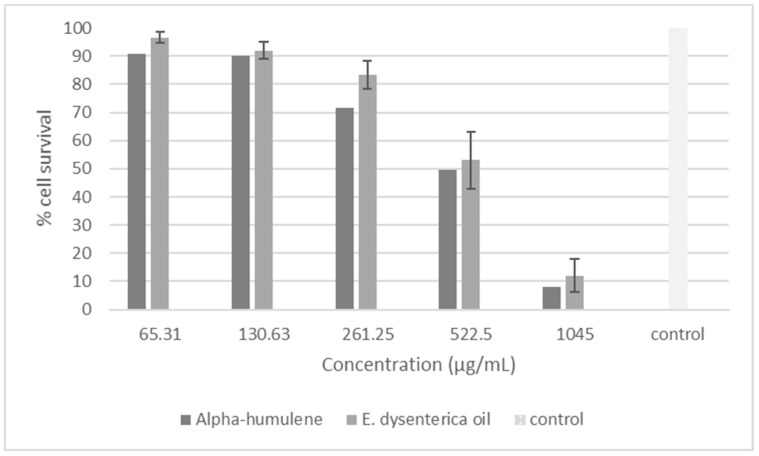
Viability of cells treated with an essential oil from the leaves of *E. dysenterica* and α humulene after 24 h of treatment in a fibroblast cell line L-929 by MTT assay. Fibroblast cell without treatment was used as control.

**Figure 3:**
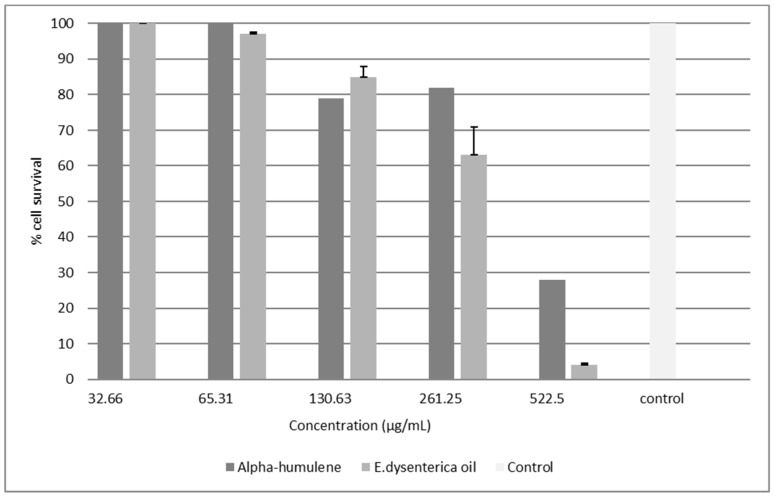
Viability of cells treated with an essential oil from the leaves of *E. dysenterica* and α humulene after 24 h of treatment in a microphages cell line RAW by MTT assay. Macrophages cell without treatment was used as control.

For L929 cells, the leaf oil showed an IC_50_ value of 542.2 μg/mL, while α-humulene presented an IC_50_ of 577.4 μg/mL, very close to each other in fact (Figure 2). The effects of the oEd and α-humulene tested on macrophages (RAW) were safe, at concentrations of 292.0 μg/mL and 405.9 μg/mL, respectively (Figure 3). The other study using *E. dysenterica* leaves, described in the literature, showed similar results to cell viability [10].

Compared with other studies, the reported results confirm that terpenes show low toxicity in human fibroblasts and in macrophages [11]. β-caryophyllene also found in *Syzygium aromaticum* oil did not show cytotoxicity to skin cells [11] α-humulene was also significantly less cytotoxic against human fibroblasts than against tumor cell lines (12). Terpenes are the main constituents of essential oils and have a ‘Generally Regarded as Safe’ (GRAS) status with the Federal Drug Administration of the United States of America. Furthermore, they have low systemic toxicity and skin irritancy, not to mention good percutaneous absorption [12,13,14]. The data above shows that sesquiterpenes are abundant in oEd, corroborating results present in the literature regarding classes of terpenes. Furthermore, a Phototoxicity Test (OECD N° 432, 2004) using 3T3 Neutral Red Uptake cells showed that hydroalcoholic leaf extract from *E. dysenterica* had a non-phototoxic potential without photo-irritation, demonstrating its safety [10]. These results suggest a potential application of *E. dysenterica* oil in skin treatment

#### 2.1.3. Scratch Wound Healing Assay

**Figure 4:**
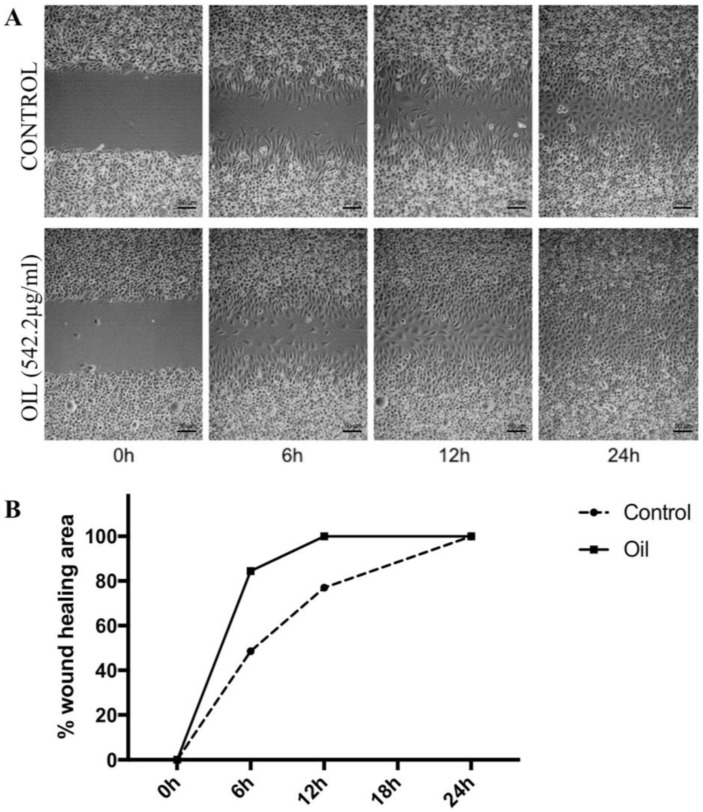
Effects of essential oil from *E. dysenterica* leaves on migration changes in L929 fibroblast cells after treatment monitored by phase contrast microscopy. A scratch wound healing assay was performed on L929 cells treated with 542.2 µg/mL of oEd to determine cell migration ability. Showing scratch wounds in L929 cells at time 0 h and representing wound status 24 h after the initiation of the scratch when the cells were treated with the vehicle control and concentration of oEd. Wounds were created and oEd was added immediately. Wounds were evaluated at 6, 12, and 24 h after oEd administration. (**A**). Wound healing area was measured by calculating the wound area in each period and expressed as a percentage of the initial wound area at time zero, with extract compared to the control (**B**). The statistical analysis (One way ANOVA with Tukey’s test) compares treatment with control (*p* < 0.005).

Pharmacological studies carried out with the oEd showed a pronounced topical healing action of this natural product. The results of the oEd and α-humulene application in the scratch assay model showed that oEd at the dose of 542.2 µg/mL (IC50) promoted fibroblast migration in mechanically induced wounds, an effect that was significantly different from the control (*p* < 0.05). (Figure 4). The complete closure of the wound was observed after 12 h of treatment, while in the control group, this complete closure was only observed after 24 h (Figure 4). Interestingly, isolated α-humulene was unable to increase cell migration (data not shown), which indicated that α-humulene and β -caryophyllene, as the major components from the *E. dysenterica* essential oil, act synergistically in stimulating skin cell migration [15].

Similar to our results, literature showed that a hydroalcoholic *E. dysenterica* leaf extract promoted cell regeneration in HFF-1 fibroblasts after UVA exposure [10]. Extracts of *Calendula officinalis* and the triterpenoid faradiol myristate have also been shown to stimulate both the proliferation and migration of fibroblasts [16].

Dermal wound healing involves a cascade of complex events including angiogenesis and extracellular matrix remodeling [17]. This mitogenic effect is a positive event for the wound healing process because fibroblasts are important cells involved in wound contraction, fibroplasias, extracellular matrix production, and the attenuation of inflammatory mediators [18]. The activity of oEd can be the result of the synergistic effects of associated compounds present [15], such as β -caryophyllene, a sesquiterpene, which is reported in the literature as possessing potential anti-inflammatory activity [19]. Research has shown the anti-inflammatory in vivo effects of β -caryophyllene and α-humulene with their ability to inhibit histamine, in a similar way to dexamethasone [19]. In addition, β-caryophyllene inhibited both interleukin IL-2 and IL-10 cytokines, suggesting that these terpenoid compounds also have an anti-inflammatory potential through the inhibition of T-cell immune responses, ratifying its immunomodulatory potential [20]. In addition, it has been shown that β-caryophyllene binds to the CB2 subtype of the cannabinoid receptors, suggesting that β-caryophyllene may be involved in the anti-inflammatory effect of oEd, by means of CB2 activation, and that this sesquiterpene could potentially be modulating the inflammatory response via the endocannabinoid system [21].

Khorshid et al. (2010) have highlighted the wound healing effects of some secondary metabolites stimulating fibroblasts. The essential oil from *Plectranthus tenuiflorus* leaves stimulates the growth of fibroblasts in vitro, similarly to this study [22]. These findings imply that the terpenes present in the leaf oil of *E. dysenterica* could be responsible for promoting wound healing activity by acting at the proliferative stage via: angiogenesis, collagen deposition, granulation tissue formation, epithelialization, and wound contraction. Sesquiterpenes are reported to facilitate wound healing. Investigation of the in vivo anti-inflammatory action of the essential oil of *Cordia verbenacea* has suggested that the active compounds α-humulene and β-caryophyllene that act with the pro-inflammatory cytokine tumor necrosis factor α (TNF-α), could be an interesting target, since this molecule is involved in the induction of inflammation and many cutaneous and systemic inflammatory diseases [19]. Evidence suggests that α-humulene might constitute a relevant alternative for the control of B1-receptor upregulation [23].

Tsala et al. (2013) reported that numerous crude natural preparations and purified compounds have been tested for their effect(s) on skin cell migration or proliferation. Of the aforementioned, the terpenoids accelerated re-epithelialization and fibrosis in an artificial dermal surgical wound and suggested the possibility of their use as topical agent modulators of skin cells: Fibroblasts and keratinocytes [24]. The sesquiterpene lactone, deoxyelephantopin isolated from *Elephantopus scaber* Linn., promoted significant wound healing activity by increasing: cellular proliferation, granulation tissue formation, collagen synthesis, and the rate of wound contraction [25]. Recently, the photoprotective effects of leaf hydroalcoholic extract were demonstrated in HFF-1 human foreskin fibroblasts. A vegetable derivative was found to promote cell regeneration after ultraviolet A (UVA) exposure [10].

#### 2.1.4. Effect of oEd, α-Humulene and Dexamethasone on NO Production

Innate immune system cells play a central role in the inflammatory response following tissue injury. Macrophages play a critical role in the response to perturbations of tissue homeostasis and participate in the onset of the inflammatory event. These cells are also crucial for the development of subsequent events, especially the resolution of inflammation [26]. Macrophages stimulated with lipopolysaccharide (LPS), a component of Gram-negative bacteria cell walls, can produce several inflammatory mediators, among them nitric oxide (NO), the most important mediator in human health and disease. However, excessive NO levels are toxic as they form free radical groups, such as superoxide, resulting in the production of toxic peroxynitrite, which can in turn trigger consequences such as inflammatory diseases [27]. Therefore, NO production may provide a measure to assess the effects of compounds on the inflammatory process. The present study examined the potential for oEd to reduce inflammation effects in LPS-stimulated RAW 264.7 murine macrophages in vitro.

**Figure 5:**
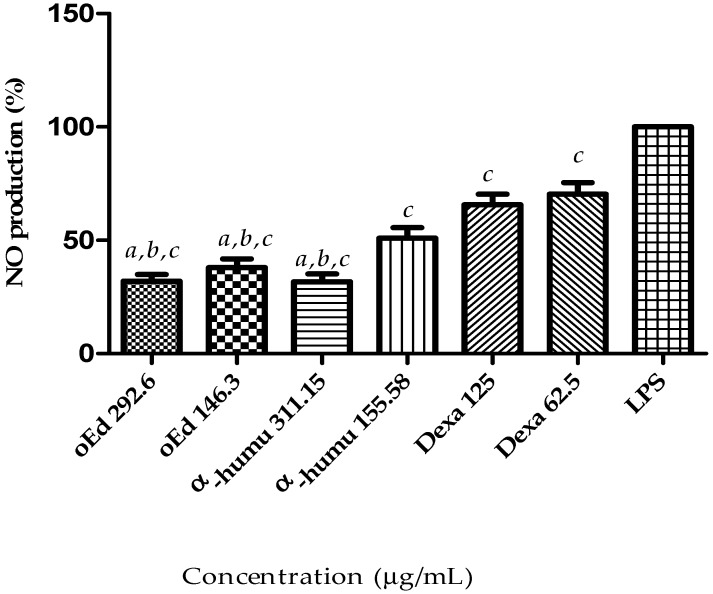
Effects of essential oil from *E. dysenterica* leaves on LPS-induced NO in RAW 264.7 using the Griess assay. Macrophages were incubated in the presence of oEd, α-humulene or dexamethasone in combination with 1 µg/mL LPS for 48 h. The significant differences related to the concentration values of essential oil (oEd); α-humulene (α-humu) and dexamethasone (Dexa) from the inhibition of nitric oxide production of following LPS-stimulation. Values in three experiments were calculated by one-way ANOVA with Bonferroni’s Multiple Comparison Test (^a^ *p* < 0.05 vs. Dexa 125. ^b^ *p* < 0.05 vs. Dexa 62.5. ^c^ *p* < 0.05 vs. LPS).

The amounts of nitrite were determined via the Griess assay [28]. We analyzed the ability of oEd to neutralize LPS. As presented in Figure 5, in stimulated RAW 264.7 cells upon LPS treatment was treated with oEd at two different concentrations (292.6 µg/mL and 146.3 µg/mL) and was observed the nitrite concentrations production in the medium decreased. The different concentrations of oEd inhibited NO production in LPS-stimulated RAW 264.7, while different concentration of dexamethasone inhibited less than 50% of NO production, used as a positive control. In addition, NO production was also significantly inhibited by α-humulene, when compared to the dexamethasone at all tested concentrations (*p* < 0.05). These findings indicate that compounds of the oEd have a pharmacological effectiveness.

It is suggested that the inhibition of the pro-inflammatory mediator NO by *Eugenia dysenterica* essential oil is due to its major α-humulene and β-caryophyllene components, as has been shown in the literature [19]. This pharmacological activity is based on: Inhibition of interleukin IL-2 and IL-10 [20], TNF α [29], histamine [19], and IL-1β [23]. Oxidative stress and inflammation are common features of many chronic diseases and their complications. They have also been linked to inflammatory disease processes. Previous studies have shown that plants possess antioxidant and anti-inflammatory properties that can help counter disease processes. Fernandes et al. (2007), reported that both compounds reduced prostaglandin E_2_ production, as well as inducing nitric oxide and cyclooxygenase (COX-2) expression in vivo, suggesting a very similar action to dexamethasone [19]. Another possibility for the anti-inflammatory action may be linked to the ability of β-caryophyllene to act as a functional CB2 receptor anti-inflammatory ligand, reducing cytokine levels in a culture of macrophages stimulated with lipopolysaccharide [21,30,31].

Recently, Han and Parker (2017) reported the anti-inflammatory action, immune-modulating, and tissue remodeling activities of *Eugenia caryophyllata* essential oil and its importance in the inhibition of pro-inflammatory cytokines in human skin disease [32]. Anti-inflammatory activity of essential oils from *Syzygium cumini* and *Psidium guajava,* other species of the Myrtaceae family, has also been observed [33]. Our group has shown a potent in vitro tyrosinase inhibitory activity of *E. dysenterica* leaf ethanol extract compared with kojic acid, exhibiting IC_50_ values of 11.88 and 13.14 mg/mL, respectively [34]. In addition, its leaves have demonstrated expressive antioxidant properties to the presence of secondary metabolites [35,36,37]. These properties indicate that *E. dysenterica* vegetable derivatives are promising constituents in developing pharmaceutical products for skin healing [10,34].

### 2.2. In Vivo Studies

#### 2.2.1. Irritation Potential of Essential Oil

The proven safety of products to the skin remains a fundamental requirement in the legislation of chemicals. The chorioallantoic membrane (CAM) assay has been used as an in vivo model for toxicology studies [38].

The safety of oEd, with regards to the irritation parameters, was shown in Table 1. When different concentrations of oEd were tested in chorioallantoic membrane, in order to determine irritancy endpoints, such as lysis, hemorrhage, and coagulation, have no adverse effects were observed indicating a minor irritant potential. However, above IC_50_ exposure, concentrations of 584 and 1168 µg/mL triggered irritancy effects such as slight and moderate vascular damage, respectively. It was observed that the positive 0.1 M NaOH control induced hyperplasia and hemorrhage in 0.5 min and coagulation in two min. The IS value of NaOH was 21, suggesting that it could induce severe irritation.

Moreira et al. (2017) evaluated the hydroalcoholic extract from *E. dysenterica* leaves using a Bovine Corneal Opacity and Permeability protocol (OECD N° 437, 2013). Results demonstrated a non-eye irritant, demonstrating that this compound is safe [10], thus corroborating our findings. In addition, Costa et al. (2015) evaluated the chitosan microparticles, which also demonstrated a non-irritating behavior [39]. 

#### 2.2.2. Angiogenesis Activity

A diversity of natural derivatives have been reported to stimulate or inhibit angiogenesis in the CAM [38].

**Figure 6:**
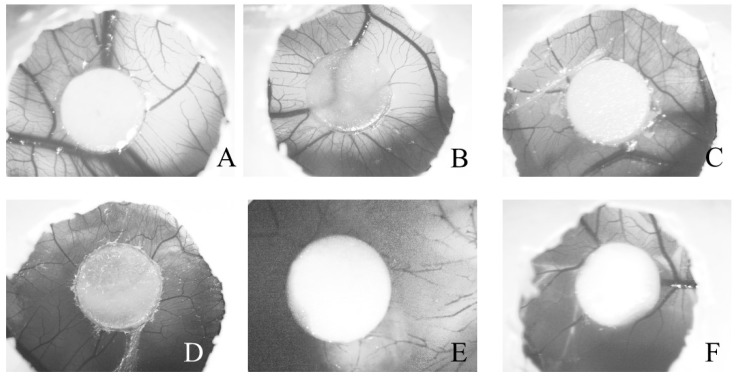
Photomicrograph of different chick embryo chorioallantoic membranes (CAM) after 72 h of treatments with (**A**), oEd 146 (oEd 146 μg/disk); (**B**), 292 (oEd 292 μg/disk); (**C**), oEd 584 (oEd 584 μg/disk); and (**D**), olive oil (20 μL, negative control); (**E**), DEXA (dexamethasone 80 μg/disk, angiogenesis inhibitor) and (**F**) RG (Regederm^®^ 20 mg/disk, angiogenesis inductor). Evaluation of CAMs showed a proangiogenic response by macroscopic semiquantitative scoring. The images illustrate representative examples of different responses (score rangeaccording to Ribatti, D, et al. 2006 [40]), as observed under an Axiovert Light Microscope with the aid of the AxioVision 100 software (both Zeiss, Göttingen, Germany), at the magnification of 0.5 × 1.25.

The results of CAM assay revealed that oil-treated CAMs branched out into more multi-stage capillaries and more abundant neo-vasculatures, as shown in Figure 6. Together, our results in the present report suggests that oEd promoted the angiogenic processes as it increased the number of blood vessels for all treatments with oEd (scoring three) equivalent to the positive control (scoring 3) in comparison to the negative control (olive oil scoring zero), which maintained the similar blood vessels number and vessel wall thickness during the experiment [40]. The inhibitor agent (dexamethasone) group presented a scarce vessel number. This effective anti-inflammatory capacity of glucocorticoid is due to the fact that drug to decrease angiogenesis is associated with significant reductions in the levels of prostaglandin E_2_ and vascular endothelial growth factor (VEGF) expression (Figure 6) [41].

Tissue repair concerns many cellular events including: Coagulation, inflammation, epithelialization, granulation tissue formation (a complex process characterized by angiogenesis), and matrix and tissue remodeling [42]. Skin repair condition is a complex reaction in the vascularized tissue associated with multifactorial origins, internal or external stimuli or both can cause the cell damage. The ultimate goal of this protective response is to rid the organism of both the initial cause of cell injury, the consequences of which can result in structural damage. However, an exaggerated or unregulated prolonged inflammatory process can also induce tissue damage and is in fact the cause of many chronic diseases [27]. Therefore, in recent years, natural compounds are being employed in wound treatments because of their ability to promote blood clotting, fight infection, and accelerate wound healing. These bioactive agents usually modulate one or more phases of the healing process [43].

According to Rojo et al. (2014), nut oil from *Pouteria lucuma* promotes wound healing by modulating inflammation with a significant decrease in the concentration of nitric oxide, cell migration, and blood vessel sprouting [44]. One pharmacological study has recently indicated that a compound from *Abelmoschus manihot* exhibits angiogenic ability using the CAM assay [45]. *Cinnamomum cassia* is another example of a medicinal plant that induces angiogenesis both in vivo and in vitro by increasing VEGF production [46].

Recently, the evaluation in vivo of Acheflan^®^ accelerates wound healing and this effect may be attributed to the compound α-humulene, which has revealed important anti-inflammatory action beyond demonstrated completely and absorbed fast when applied topically. This result showed higher topical effectiveness, probably due to its involvement with the increase angiogenesis and dermal remodeling imputed to the α-humulene and activation of growth factor VEGF [47,48]. Innumerable angiogenic factors such as VEGF, TNF, IL-6, and IL-8 are activated by inflammatory cells and the VEGF family members are the major mediators of the regulatory machinery that controls angiogenesis during development and in pathological conditions [17]. The widespread dissemination of cannabinoid receptors agonists on skin in mast cells provides implications for an anti-inflammatory property and their putatively broad therapeutic potential [49].

These results demonstrate a positive effect of essential oil on the wound healing process in the L929 fibroblasts cell line, probably due to NO inhibition and its association with the increase in the angiogenic process activities exhibited by phytochemicals in the essential oil from *E. dysenterica* leaves.

Based on the aforementioned bioactivities, it might partially explain why this oil can exhibit the anti-inflammatory effect in the LPS-stimulated RAW246.7 macrophages and the scratch-induced wounds. Our results suggest that this native Brazilian species is capable of neutralizing cytokines and exhibits good anti-inflammatory activity in vitro, a fact that seems to relate to mediator modulation by phytochemicals. Our analyses showed that this species contains high concentrations of bioactive sesquiterpene compounds, such as β-caryophyllene and α-humelene, thus suggesting that these compounds may be responsible for their activities. The results indicate that essential oils from *E. dysenterica* may be used in future as an alternative in potential new wound healing applications.

## 3. Materials and Methods

### 3.1. Material

Dulbecco’s modified eagle medium (DMEM) was purchased from Gibco (Glasgow, UK). The L-929 and RAW 264.7 cell lines originated from the American Type Culture Collection (ATCC, Manassas, VA, USA) from the Adolf Lutz Institute Cell Bank. Dexamethasone, fibronectin, pure sesquiterpene (α-humulene), and (3,[4,5-dimethylthiazol-2-yl]-2,5-diphenyltetrazolium bromide (MTT) were supplied by Sigma-Aldrich (St. Louis, MO, USA). All other solvents and reagents were of analytical grade and provided by Sigma-Aldrich (St. Louis, MO, USA). All assays were performed using ultrapure water (Milli-Q Millipore, Burlington, VT, USA). The fertilized chicken eggs were donated from poultry trader Bonasa (Federal District, Brasilia, Brazil) and were used according to the protocols approved by the Animals Ethics Committee of the University of Brasília (Process No. 77/2017).

### 3.2. Plant Material

*Eugenia dysenterica* leaves were collected in Brasilia (Federal District, Brasilia, Brazil) by Prof Dr Sueli Maria Gomes. Following botanical identification, a voucher specimen was deposited in the Herbarium of University of Brasilia (UB) under the registration identification code UB 914.

### 3.3. Preparation of Essential Oil from E. dysenterica Leaves

Hydrodistillation of fresh *E. dysenterica* leaves was carried out in a Clevenger apparatus (Biogenic, Brasilia, Brazil). The process was continued for 4 h after the appearance of the first drop of distillate [50]. The oil yield was calculated on a dry-weight basis (*v*/*w*). The harvested essential oil was stored at −20 °C prior to the biological study.

### 3.4. Analysis of Essential Oil from E. dysenterica Leaves Using GC-MS

The GC analysis was performed on a Shimadzu gas chromatograph-model QP2010 Ultra (Kyoto, Japan). The oil was separated on a Rtx-5MS capillary column (30 m × 0.25 mm × 0.25 μm, Restek, Lisses, France). The column temperature program was: 50 °C/1.5 min, followed by a rate of 4 °C·min^−1^ to 200 °C, then a rate of 10 °C to 300 °C, and finally 300 °C/5 min. The injector temperature was 280 °C. The sample (1.0 μL in ethyl acetate) was injected with a 1:30 split ratio. The carrier gas was helium at 1.0 mL/min. Data processing of the obtained mass spectra was carried out with the GC-MS Solution Ver. 4.45 (Shimadzu Corporation, Kyoto, Japan). The identification of essential oil compounds was assigned by comparison of their retention time using caryophyllene oxide (Sigma-Aldrich, St. Louis, USA and a-humulene (Sigma-Aldrich, St. Louis, MO, USA) used as a reference standard, as well as by comparison of the mass spectra fragmentation patterns with the NIST—Mass Spectral Database, version 2.2, 2014 (NIST Standard Reference Data, USA).

### 3.5. Cytotoxicity—Tetrazolium-Based Colorimetric Assay (MTT)

A mouse fibroblast cell line (L929) and macrophages cell line (RAW 264.7) were cultured in DMEM supplemented with 10% fetal bovine serum and 1% antibiotics (penicillin-streptomycin). Samples were maintained at 37 °C and 5% CO_2_ in a humidified atmosphere for 72 h. Fibroblast cell lines were detached with trypsin (0.25%) ethylenediaminetetraacetic acid (EDTA; 1 mM) solution and RAW 264.7 with a cell scraper. The cytotoxicity assay was performed to determine the mitochondrial viability of L-929 and Raw cell lines after treatment with the essential oil [51]. The L-929 cell suspension was prepared from confluent monolayer cultures and plated at a density of 1.5 × 10^4^ cells, while the RAW cell line (2 × 10^4^ cells) were aliquoted into each well of a 96-well microtitre plate. Plates were incubated overnight at 37 °C in a 5% CO_2_ incubator. Cells were subsequently treated with 200 µL of the appropriate dilutions of the previously prepared oil in growth medium with 0.5% DMSO in concentrations ranging from 32.656 to 4180 µg/mL for oil, or 34.727 to 4445 µg/mL to α-humulene. DMEM only, or with 0.5% DMSO, were used as positive and negative controls, respectively. All samples were incubated in parallel with the test cultures for 24 h. After incubation, the supernatant was aspirated and 50 µL of MTT solution at 1 mg/mL in phosphate-buffered saline (PBS) was added to each well. Plates were incubated for a further 4 h. Acidified isopropanol (150 µL) was then added to solubilize the formazan crystals produced by mitochondrial activity. Cell survival was quantified by an absorbance measurement at 570 nm in a Beckman Coulter Microplate reader (Indianapolis, IN, USA). The IC_50_ value represents the sample concentration required to inhibit 50% of cell survival.

### 3.6. Scratch Wound Healing Assay

A wound healing assay was performed to observe the effect of the oED on the migration ability of the epithelial cells. The L929 cells were seeded at 1 × 10^6^ cells/well into fibronectin-coated six-well plates. The cells monolayer was manually scratched with a yellow PBS-rinsed plastic pipette tip and treated with 542.2 µg/mL solution of oil plus DMEM, or only with DMEM (control) as well as α-humulene (biological standard). Each evaluated sample was plated in triplicate. An inverted microscope (Zeiss Primo Vert, Göttingen, Germany) equipped with a digital camera (Zeiss ERc 5s, Göttingen, Germany) was used to obtain images of the wound healing at different timepoints of each treatment under magnification (×10). Wound closure was monitored at 24 h, until the borders of the wound could no longer be identified. The closure was measured by wound area in each period and expressed as a percentage of the initial wound area at 0 h. Statistical tests were considered as significance at the level of 5% (*p* < 0.05).

### 3.7. Nitrite Assay

Analysis of the intracellular nitric oxide production by macrophages was performed using a protocol based on Griess’s method [28]. Firstly, the MTT assay was undertaken to determine the non-cytotoxic concentrations of oil that maintained cell viability ≥ 50% (IC_50_). Briefly, the cells (0.3 × 10^6^ cells/mL) in 10% FBS–DMEM with phenol red were seeded into 12-well plates and incubated overnight at 37 °C. Samples were subsequently exposed to two different oil concentrations (146.3 and 291 μg/mL), α-humulene (155.58 and 311.15 μg/mL) or culture medium only (control cells) for 3 h at 37 °C and stimulated with 1 μg/mL LPS for 48 h. The cell free culture medium was collected and analyzed for nitrite accumulation as an indicator of NO production by the Griess reagent [28] as follows: 100 μL of Griess reagent (0.1% naphthyl ethylenediamine and 1% sulfanilamide in 5% H_3_PO_4_ solution) were added to 100 μL of each supernatant from the sample-treated cells. The plates were incubated for 5 min at 37 °C and subsequently read at 540 nm. The quantity of nitrite in the supernatant samples was estimated by measuring the absorbance of a standard solution of sodium nitrite salt treated under the same conditions with Griess reagent. The percentage of inhibition was expressed as 100 × [1-(NO release with sample-spontaneous release)/(NO release without sample-spontaneous release)].

### 3.8. HET-CAM Test

#### 3.8.1. Evaluation of the Irritation Potential

The Hen’s Egg Test-Chorioallantoic Membrane (HET-CAM), was used to determine the essential oil irritation potential, as previously described [52]. The SPF (specific pathogen free) eggs were incubated for 10 days at a temperature of 38.0 ± 0.5 °C and a relative humidity of approximately 60–70%. A 300 μL aliquot of each sample at different concentrations (a range of 73–1168 µg/mL) eluted in olive oil was applied to five different fertilized eggs per group. After 20 s of contact time, the sample was removed, and the membrane washed with an isotonic saline solution at 37 °C. The CAM reactions were observed for 300 s. At 0.5, 2, and 5 min, the following phenomena were observed: Hyperplasia, hemorrhage, and coagulation (maximum value of 21). Regarding the irritation score (IS), 0–0.9 represents no irritation, 1–4.9 represents slight irritation, 5–8.9 is considered as moderate irritation, and 8–21 constitutes serious irritation [51]. The membrane was examined over a five-minute period. All reactions observed were scored according to the time of their appearance and were analyzed for hemorrhage, lysis and coagulation of blood vessels. The products were classified according to their irritancy potential using the HET-CAM test and the final classification. The final result was expressed as the mean value of five egg tests. The positive control was 0.1 M NaOH. The negative control was 0.9% NaCl and olive oil. The HET-CAM test was performed using five eggs per sample.

#### 3.8.2. Chicken Chorioallantoic Membrane (CAM)

The chicken chorioallantoic membrane (CAM) assay was performed according to Melo Reis et al. (2010) [53]. After incubation at 37 °C in a humidified atmosphere (60–70% relative humidity) with appropriate rotation, fertilized eggs were opened, and the shell membrane removed to expose the CAM. On day five of incubation, a circular window was opened in the large end of the eggshell and the membrane removed. The window was resealed with adhesive tape and eggs were returned to the incubator until day eight. Filter paper disks soaked with 20 μL of *E. dysenterica* oil solutions at different dilutions (ranging from 73–1168 µg/disk) were placed on top of the growing CAM at day 13 of incubation under sterile conditions. Positive (Regederm, Pele nova Biotecnologia, Ribeirão Preto, Brazil) 20 μL of an aqueous solution of 1000 mg/mL (20 mg/disk), negative (20 μL olive oil), and inhibitor (80 μg/disk dexamethasone, Sigma-Aldrich, St. Louis, MO, USA) controls were included. All windows were sealed with transparent adhesive tape and the eggs were incubated at 37 °C for three days. The angiogenic response was evaluated 72 h after the treatments. On the 16th day of fertilization, the sealing tape was removed and the representative images of the control groups and or treated with the above concentrations of each of the compounds were captured by an Axiovert Light Microscope (Zeiss, Göttingen, Germany), with the aid of the AxioVision Imaging System (Carl Zeiss Microscopy, Göttingen, Germany) at a magnification of 0.5 × 1.25. Blood vessels were quantified by macroscopic evaluation of the angiogenic response by analyzing variations in the distribution and density of CAM vessels adjacent to the graft site. Based on the semiquantitative scoring method by Reference [40], the arbitrary scale of zero–five values is as follows—0 describes a condition of the vascular network that is unchanged with respect to the time of grafting; 1 marks a slight increment in vessel density associated with occasional changes in the course of vessels converging toward the graft site; and 2, 3, 4, and 5 correspond to a gradual increase in vessel density associated with increased irregularity in their course; while a rating of **5** also highlights strong hyperemia [40].

### 3.9. Statistical Analysis

For the cytotoxicity assay, statistical analysis was performed using the mean values of at least three independent replicates using GraphPad Prism version 7.00 for Windows, GraphPad Software, La Jolla California USA by applying the log (inhibitor) vs. response—Variable slope analysis. For the Scratch assay, statistical analysis was performed using the Two-way ANOVA followed by Tukey’s multiple comparisons test (using GraphPad Prism version 7.00 for Windows, GraphPad Software, La Jolla, CA, USA) with a significance level of 5% (*p* ˂ 0.05). All data are presented as mean values with the corresponding standard deviation indicated (mean ± SD).

## 4. Conclusions

The present study proved that the highly active phytochemicals containing sesquiterpenes, present in the essential oil extracted from *E. dysenterica* leaves, may serve as a potent natural wound healing compound with an important role in human health and the toxicological evaluation of oEd. Our data demonstrated that oEd provides stimulation for the migration in epithelial cells. This action is probably due to the presence of terpene compounds in its composition. In addition, it affords anti-inflammatory protection by suppressing the production of excess intracellular nitric oxide. Moreover, toxicological evaluation of oEd provides us with crucial information, which is helpful in understanding its effect(s) in an in vivo system. The results not only suggested that oEd has the potential to be used in complementary and alternative medicine for the treatment of skin injuries, they also support its feasibility to obtain effective and safe dermatological products for the topical treatment of skin injuries. Our results provide a starting point for further studies aimed at elucidating the molecular processes and signaling pathways underlying fibroblast proliferation and migration induced by *Eugenia dysenterica* preparations.

## Figures and Tables

**Table 1:** The effects irritation score (IS) of essential oil from *Eugenia dysenterica* leaves in the HET-CAM assay.

Samples	Hyperemia	Hemorrhage	Coagulation	IS
Olive oil	0	0	0	0
0.9% NaCl	0	0	0	0
73 µg/mL	0	0	0	0
146 µg/mL	0	0	0	0
292 µg/mL	0	0	0	0
584 µg/mL	0	3	0	3
1168 µg/mL	0	5	0	5
0.1 M NaOH	5	8	8	21
